# MFE-DDI: A multi-view feature encoding framework for drug-drug interaction prediction

**DOI:** 10.1016/j.csbj.2025.05.029

**Published:** 2025-05-26

**Authors:** Lingfeng Wang, Yinghong Li, Yaozheng Zhou, Liping Guo, Congzhou Chen

**Affiliations:** a Beijing University of Chemical Technology, Beijing, 100029, China; b China Japan Friendship Hospital, Beijing, 100029, China

**Keywords:** Drug interaction, Multi-view drug features, Multidimensional feature fusion, Deep learning

## Abstract

Multidrug combination therapy has long been a vital approach for treating complex diseases by leveraging synergistic effects between drugs. However, drug-drug interactions (DDIs) are not uniformly beneficial. Accurate and rapid identification of DDIs is critical to mitigate drug-related side effects. Currently, many computational-based methods have been used to expedite the prediction of DDIs. However, most of these methods use a single perspective to obtain drug features, which have limited expressive capabilities and cannot fully represent the essential attributes of drugs. In this study, we propose the Multi-view Feature Embedding for drug-drug interaction prediction (MFE-DDI), which integrates SMILES information, molecular graph data and atom spatial semantic information to model drugs from multiple perspectives and encapsulate the intricate drug information crucial for predicting DDIs. Concurrently, the feature information extracted from different feature encoding channels is fused in the attention-based fusion module to fully convey the essence of drugs. Consequently, this approach enhances the efficacy of the DDI prediction task. Experimental results indicate that MFE-DDI surpasses other baseline methods on three datasets. Moreover, analysis experiments demonstrate the robustness of the model and the necessity of each component of the model. Case studies on newly approved drugs demonstrate the effectiveness of our method in real scenarios. The code and data used in MFE-DDI can be found at https://github.com/2019040445/MFE_DDI.

## Graphical abstract

**Figure:**
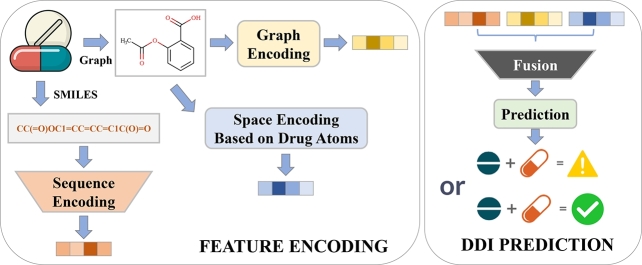

## 1 Introduction

The combined use of drugs is a common approach for managing complex or co-existing conditions by leveraging the synergistic effects of different drugs. For example, in the treatment of pulmonary tuberculosis, triple therapy typically includes isoniazid, rifampin, and pyrazinamide, while quadruple therapy adds ethambutol to enhance treatment efficacy and prevent drug resistance [1]. However, due to the varied structures and chemical properties of drugs, using drug combinations also has the potential hazards of adverse drug-drug interactions (DDIs) [2], [3]. Therefore, prompt detection of unexpected DDIs is imperative.

In recent years, deep learning-based drug relation extraction techniques [4], [5], [6] have enabled the automatic identification of known DDIs from text. However, such methods heavily rely on existing documented literature and struggle to discover potential interactions. Therefore, developing efficient computational DDIs prediction approaches has become a crucial complement to traditional text-mining techniques.

Numerous computational approaches have been formulated nowadays to detect DDIs. In general, these technologies can be divided into three categories: sequence-based, molecular graph-based and knowledge graph-based.

Traditional DDIs prediction models mostly use sequence data such as chemical fingerprint and SMILES as input [7], [8], [9], [10]. Fingerprint-based methods predict DDIs using fingerprint vectors, which encapsulate critical drug characteristics, including substructures, target relationships, and potential side effects. However, they face scalability issues due to limited applicability of certain fingerprints and fail to show the intuitive spatial structure of drugs no matter how many fingerprints are integrated. In addition, many sequence-based methods used SMILES to represent complex drugs. For example, Ryu et al. [7] used SMILES sequences to extract drug representations, calculated the similarities between drugs to construct structural feature vectors, and subsequently employed deep neural networks for prediction. DeepPurpose [11] integrates SMILES processing through an array of neural architectures, including Convolutional Neural Networks (CNN) [12], Recurrent Neural Network (RNN) [13], [14], and Transformer [15], to enhance feature extraction for DDI prediction. Despite these advances, SMILES primarily represents the one-dimensional structure of a drug, which may not sufficiently capture the complex spatial conformations of molecules.

By directly representing molecular structures as graphs with atoms as nodes and bonds as edges, the molecular graph-based methods [16], [17], [18], [19], [20], [21] compensate the critical shortcomings of sequence-based representations. Techniques such as graph convolutional neural network (GCN) [22], [23], graph attention network (GAT) [24], [25], and gated graph neural network [26], [27] are employed to delineate the structural characteristics of molecules. Innovatively, SA-DDI [16] utilizes a substructure-aware graph neural network to adaptively discern substructures of varying dimensions and configurations, analyzing their interactions to pinpoint crucial substructures for DDI prediction. Furthermore, 3DGT-DDI [17] integrates a pre-trained text attention mechanism to extract relational data from textual descriptions, enhancing predictions through molecular structures and spatial analysis to better understand substructural influences on DDI dynamics. DeepGCL [18] employs dual-graph contrastive learning, using GCNs to encode both molecular structures and drug-pair subgraphs while maximizing feature consistency. While these graph-based models adeptly capture structural characteristics of molecular graphs, incorporating spatial conformations would further enhance their representational sufficiency.

Knowledge graph-based methods [28], [29], [30], [31], [32], [33] garner extensive biomedical information to predict DDIs by constructing heterogeneous graphs between drugs and other entities related to drugs, such as proteins, genes, etc. Chen et al. [30] proposed MUFFIN, which used drug molecular structure and biomedical knowledge graph to predict DDIs. Wang et al. [33] extracted drug features from the two dimensions of drug graph structure and knowledge graph, and then designed a novel feature fusion method to further predict DDIs. By utilizing multi-biological entity information, the enhanced expression of drug characteristics improved DDIs prediction performance to some extent. However, excessive reliance on external biological entities may obscure the essential characteristics of drugs, ultimately compromising prediction accuracy.

As the physicochemical properties of drugs are inherently complex, it is imperative to comprehensively extract multi-view information to elucidate the essence of drugs. SMILES represents the one-dimensional structure of a drug without spatial information. Although molecular graphs add structural details, the lack of spatial conformations makes them insufficient.

In this article, we introduce Multi-view Feature Embedding for drug-drug interaction prediction (MFE-DDI), which incorporates the SMILES and 2D molecular graph of the drug, alongside semantic information including the atomic spatial structure derived from drug space encoding, to extract more comprehensive drug features. By encoding the drug molecular graph using atomic centrality encoding, spatial encoding and so on, the finer-grained atomic spatial semantic features of drugs can therefore be further characterized. To the best of our knowledge, our MFE-DDI method represents the first to integrate SMILES, molecular graph, and spatial semantic information for DDI task. It fully leverages multidimensional information to extract drug features, thereby providing more precise and effective input for downstream prediction tasks.

Our model architecture integrates three distinct encoding channels to analyze drug features comprehensively. The first channel is a one-dimensional sequence feature encoder that employs the FCS algorithm [34] to decompose the SMILES sequence into shorter substructures, utilizing a Transformer to encode these sequence characteristics. The second channel, a molecular graph feature encoder, leverages the Message Passing Attention Network (MPAN) [35] to extract structural details from graph representations of molecules. The third channel incorporates atomic centrality, spatial and edge encoding, to delineate finer-grained atomic and spatial conformations along with semantic characteristics of the drug. Ultimately, the feature vectors from these three channels are amalgamated in the attention-based feature fusion module to efficiently fuse different features and subsequently inputted into the prediction module to obtain the drug-drug interaction scores.

Overall, the main contributions are summarized as follows:

(1) MFE-DDI employs a three-channel feature encoder to extract multidimensional features of drugs, including drug SMILES sequence features, molecular graph structure features and finer-grained atomic spatial conformation features. This approach enables a more precise representation of drugs.

(2) MFE-DDI utilizes a feature fusion method based on attention mechanism to integrate drug features across multiple dimensions, thereby augmenting the efficacy of downstream prediction tasks.

(3) Experiments on three datasets and other analytical validation demonstrate our method possesses significant effectiveness in predicting drug-drug interactions.

## 2 Methodology

**Fig. 1:**
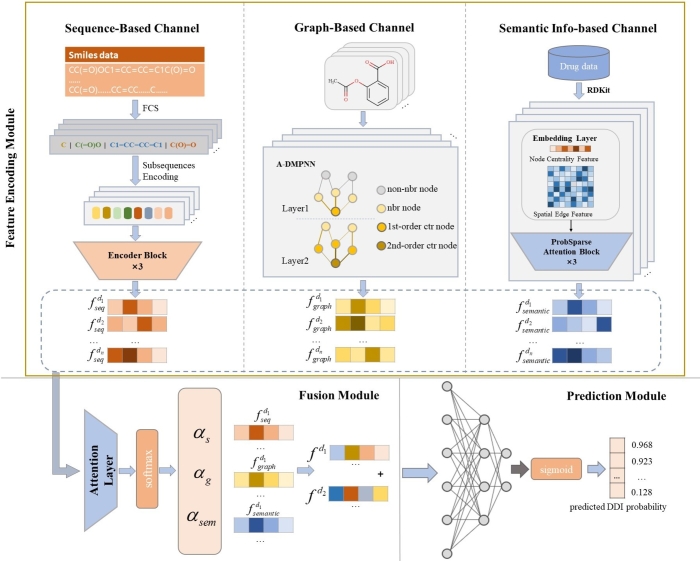
The overall framework of MFE-DDI. The feature encoding module contains three channels to obtain different view of features. The multidimensional feature decoder contains feature fusion module based on attention mechanism and prediction module with three linear layers to obtain the final scores of drug-drug interactions.

This section will introduce the methods used in the MFE-DDI model. The first is multidimensional feature encoding module. The SMILES characteristics information of drugs is obtained through the SMILES feature encoding module; the graph feature of drugs is obtained through the Graph encoder; the atom semantic feature encoding module is employed to obtain the atomic spatial feature of drugs. Upon feature extraction, a feature fusion module based on attention mechanism is adapted to integrate multidimensional drug features comprehensively. Subsequently, the amalgamated feature vector is channeled into the prediction module to compute DDI scores. The entire framework is depicted in Fig. 1.

In DDI prediction task, the drug set containing *n* drugs is represented as $D=\{d_{1},d_{2},...d_{n}\}$; The task of predicting drug-drug interactions (DDIs) is framed as a binary classification problem. This can be mathematically represented by the function f:D×D→[0,1], where $f(d_{i},d_{j})=1$ signifies the presence of an interaction between drugs $d_{i}$ and $d_{j}$, and $f(d_{i},d_{j})=0$ denotes no interaction.

### 2.1 SMILES sequence-based feature encoding module

The SMILES sequence of drugs consists of characters for atoms and bonds, most of which represents one atom as one character (a few atoms are composed of multiple characters, such as chlorine (Cl), bromine (Br), lead (Pb), etc.). Many methods directly model the correlation between an atom and another, and generate deep representations of the drug based on the correlation to predict drug-drug interactions. However, the relative position information between individual atoms is not explicitly enough to characterize chemically meaningful groups in a drug sequence. Therefore, character-level encoding methods are not sufficient to encode drug sequences, and our method more considers the relationship between character groups, which are expressed as common chemical structures, such as chemical groups.

In the first channel of our model, we use the FCS algorithm [34] to decompose the SMILES sequence into common subsequences to obtain medium-sized substructures. These substructures are granular units that can express biochemical semantics relatively completely. These substructures are then encoded, through which the information encoding of a single atomic symbol or bond is converted into an encoding of substructures composed of multiple atoms and bonds. This can better reflect the role of important functional groups of drug molecules in DDIs prediction. For the sequence information of drug $d_{i}$, its substructure sequence can be obtained through the FCS algorithm:

(1) $$X_{d_{i}}=\text{FCS}(d_{i})=\{d_{i_{1}},d_{i_{2}},...,d_{il}|d_{i_{k}}\in V\}$$

where *V* is the FCS vocabulary; $d_{i_{k}}$ is the subsequence of drug $d_{i}$ encoded by FSC; the parameter *k* is defined to limit the maximum length of the input drug subsequences. When an input sequence exceeds this length, the extra part will be discarded. When less than *k*, the missing parts will be filled with zeros.

After deriving subsequences, we employ the Transformer-based self-attention network [15] to perform deep encoding and extract comprehensive sequence feature vectors for drugs. Transformer is a well-known model in the field of natural language processing that uses the mechanism of attention to evaluate contextual features in text sequences. Its multi-head attention feature allows the model to capture diverse features and interactions from various subsequences, effectively producing embeddings that reflect contextual information. These subsequences, processed through the FCS algorithm, are fed into the Transformer to generate a feature vector $f_{\text{seq}}^{d_{i}}$, representing the sequence structure of the drug.

(2) $$f_{\text{seq}}^{d_{i}}=\text{Transformer}(X_{d_{i}})$$

### 2.2 2D graph-based feature encoding module

In the second channel of our model, each sequence of a drug $d_{i}\in D$ is converted into a 2-dimensional molecular graph $g_{i}\in G_{\text{drug}}$, where $g_{i}=(V,E)$ with *V* denoting the set of atoms and *E* denoting the set of chemical bonds. This graph is then input into the graph encoder, which utilizes the Message Passing Attention Network (MPAN) [35] to produce the two-dimensional atomic graph feature vector for each drug. The graph feature encoder operates in the following two stages:

**Phase 1: Message Passing.** Initially, each node is set up with its intrinsic properties. The initial information of each node, denoted as $h_{v}^{0}\in R^{r}$, encapsulates the chemical characteristics of the respective atom. Subsequently, each node transmits its own information, encapsulated as message vectors, to adjacent nodes via the graph's edges. Concurrently, the node updates its hidden features by aggregating these received messages from neighboring nodes and edges, thereby refining its representation within the network. To emphasize the significance of certain neighboring nodes that contribute critical information, an attention mechanism is integrated into the feature update process. This mechanism assigns attention scores to neighboring nodes, serving as weight coefficients during the aggregation of message vectors. The computation of the aggregated message vector $m_{v}^{k}$ at the *k*-th layer is defined by the following steps:

(3) $$m_{v}^{k}=\text{Aggregation}(h_{v}^{k},e_{v,w}|w\in N(v),e_{v,w}\in E)$$

where Aggregation denotes the message aggregation function, N(v) represents the neighboring nodes of node *v*, and $e_{v,w}$ symbolizes the edge between nodes *v* and *w*. Each node then utilizes the current hidden feature $h_{v}^{k}$ and the message $m_{v}^{k}$ from a neighboring node to update its own feature, following the formula:

(4) $$h_{v}^{k+1}=\text{Update}(h_{v}^{k},m_{v}^{k})$$

where the Gated Recurrent Unit (GRU) [36] is employed as the Update function.

**Phase 2: Readout.** After *K* iterations of message passing, each node updates its hidden features by receiving message vectors from its *K*-th neighbor, resulting in the updated feature $h_{v}^{K}$. Following this, a readout function aggregates these updated features to generate a comprehensive representation of the entire graph.

(5) $$f_{\text{graph}}^{d_{i}}=\text{Readout}(h_{v}^{K},h_{v}^{0}|v\in g_{i})$$

Specifically, self-attention graph pooling is implemented as the readout function. The global representation of a drug graph is derived by aggregating the node embeddings, weighted by their respective importance scores as the following formula:

(6) $$f_{\text{graph}}^{d_{i}}=\sum_{v\in g_{i}}\alpha_{v}⊙fnn\left(h_{v}^{K},h_{v}^{0}\right)$$

In the above formula, $\alpha_{v}$ is defined as:

(7) $$\alpha_{v}=\frac{\exp(gnn(h_{v}^{K},h_{v}^{0}))}{\sum_{w\in g_{i}}\exp(gnn(h_{w}^{K},h_{w}^{0}))}$$

where $\alpha_{v}$ represents the importance score of node *v*, *fnn* and *gnn* are types of feedforward neural networks, ⊙ denotes the Hadamard product, and (,) is used to describe a connecting operation within this context.

### 2.3 Atom semantic information-based feature encoding module

In the third channel of the model, we use Molormer [37] to extract atomic features, atomic centrality encoding, spatial encoding, and edge encoding in the molecular graph to obtain the semantic feature of spatial conformation, which is based on the importance of different atoms and edges in the molecule structure. For drug $d_{i}$, the atom *x* is expressed as

(8) $$drug_{x}^{d_{i}}=[\alpha 1,\ldots ,\alpha 9].$$

In this framework, each element encapsulates various chemical properties including the number of atoms, chirality information, atomic degree, formal charge, the count of bonded hydrogen atoms, the number of free radical electrons, and the type of hybridization. Additionally, it indicates whether an aromatic bond is present and if the structure is part of a ring. All these attributes are accessible via RDKit [38] and are encoded as integers using a pre-defined dictionary.

The centrality encoding of an atom expresses the centrality and importance of the atom by utilizing the degree information of the atom. If $I_{x}^{d_{i}}$ and $O_{x}^{d_{i}}$ are the in-degree centrality metric and out-degree centrality metric of atom *x* respectively, the embedding equation of $E_{x}^{d_{i}}$, the centrality feature of atom *x* in drug $d_{i}$, is as follows:

(9) $$E_{x}^{d_{i}}=W_{\text{Siamese}}drug_{x}^{d_{i}}+W_{\text{out}}O_{x}^{d_{i}}+W_{\text{in}}I_{x}^{d_{i}}$$

where $W_{\text{Siamese}}drug_{x}^{d_{i}}$ is the atomic characteristics of atom *x* in drug $d_{i}$, and $W_{\text{out}}O_{x}^{d_{i}}+W_{\text{in}}I_{x}^{d_{i}}$ represents its centrality feature. The corresponding parameters for the other drug, $d_{j}$, in a drug pair are handled similarly. The weight matrices $W_{\text{Siamese}}$, $W_{\text{out}}$, and $W_{\text{in}}$ are learnable and shared between both drugs $d_{i}$ and $d_{j}$ within the Siamese network architecture [39]. These matrices are integral in learning the atomic characteristics pertinent to each drug.

The spatial position of atoms and bonds also has a crucial impact on the properties of molecules, so it is necessary to encode the feature of bonds. Edge e(x,y) is represented by the bond type, stereochemical bond, and the information of whether the bond is conjugated obtained through RDKit. To depict the positional relationship between two nodes within a spatial structure, we utilize the shortest path distance. For two connected nodes (x,y), the shortest path distance is assigned as the spatial position s(x,y) of the edge e(x,y). Conversely, if nodes (x,y) are not connected, the spatial position s(x,y) of the edge e(x,y) is set to −1. Upon determining the representation of the edge and its spatial structure, the embedding of edge e(x,y) for drug $d_{i}$, which includes spatial structure information, is defined as follows:

(10) $$E_{\left(x,y\right)}^{d_{i}}=\frac{1}{k}\sum_{1}^{k}P_{l}^{d_{i}}W_{\text{edge}}+W_{\text{spat}}s_{x,y}^{d_{i}}$$

In this formula, $P_{l}^{d_{i}}$ represents the *l*-th edge on the shortest path between atoms *x* and *y*, while s(x,y) captures the spatial positioning of the edge e(x,y). The term *k* quantifies the total edges on the shortest path $P_{l}^{d_{i}}$. To encode these spatial and structural relationships, we deploy shared weight matrices $W_{\text{edge}}$ and $W_{\text{spat}}$, which are adaptable through learning. This encoding strategy is uniformly applied across all corresponding parameters for each drug $d_{i}$.

The encodings for atomic features, atomic centrality, spatial details, and edges are collectively processed to distill comprehensive drug characteristics through an encoder using ProbSparse self-attention [39]. These detailed features are labeled as $f_{\text{semantic}}^{d_{i}}$:

(11) $$f_{\text{semantic}}^{d_{i}}=\text{ProbSparse}(E_{\text{atom}}^{d_{i}},E_{\text{edge}}^{d_{i}})$$

At this point, we have obtained more detailed semantic information about the spatial structure of atoms.

### 2.4 Multidimensional feature decoder and DDI prediction

This module performs multidimensional feature fusion on the feature information obtained from the above three modules based on an attention mechanism. The critical information is fused to assign learnable weights. Then the overall features are input to the decoder for DDI prediction. Given the obtained $f_{\text{seq}}^{d_{i}}$, $f_{\text{graph}}^{d_{i}}$, and $f_{\text{semantic}}^{d_{i}}$, the attention mechanism is calculated as follows:

(12) $$\left(\alpha_{\text{s}},\alpha_{\text{g}},\alpha_{\text{sem}})=\text{attention}(f_{\text{seq}}^{d_{i}},f_{\text{graph}}^{d_{i}},f_{\text{semantic}}^{d_{i}}\right)$$

where $\alpha_{s}$, $\alpha_{g}$ and $\alpha_{sem}$ denote the attention coefficients of embedding $f_{\text{seq}}^{d_{i}}$, $f_{\text{graph}}^{d_{i}}$, and $f_{\text{semantic}}^{d_{i}}$ respectively.

Supposed that the embedding vector $f_{\text{graph}}^{d_{i}}$ is represented as *g*. We first apply the nonlinear transformation and then multiply it by the shared attention vector $W_{2}$ to obtain its attention value w as follows:

(13) $$w_{\text{g}}=\tanh(W_{1}⊙g+b)⊙W_{2}$$

where $W_{1}$ is the weight matrix, *b* is a bias vector and $W_{2}$ is a shared attention vector. The same are $w_{\text{s}}$ and $w_{\text{sem}}$.

Then, we use the softmax function to regularize the values. It ensures that the sum of these coefficients is equal to 1, so they indicate the relative importance of different features:

(14) $$\alpha_{\text{g}}=\text{soft}(w_{\text{g}})=\frac{e^{w_{\text{g}}}}{e^{w_{\text{s}}}+e^{w_{\text{g}}}+e^{w_{\text{sem}}}}$$

Similarly, $\alpha_{\text{s}}=\text{soft}(w_{\text{s}})$, $\alpha_{\text{sem}}=\text{soft}(w_{\text{sem}})$. Finally, the overall drug embedding $F^{d_{i}}$ is obtained as

(15) $$F^{d_{i}}=\alpha_{\text{s}}⊙f_{\text{seq}}^{d_{i}}+\alpha_{\text{g}}⊙f_{\text{graph}}^{d_{i}}+\alpha_{\text{sem}}⊙f_{\text{semantic}}^{d_{i}}$$

In the prediction process, the combined multi-dimensional features of each drug pair are input into a three-layer fully connected decoder. The output is processed through a sigmoid function, which estimates the probability of drug-drug interaction (P∈[0,1]). As DDI prediction fundamentally constitutes a binary classification task, we utilize the binary cross-entropy loss function:

(16) $$L=-\frac{1}{N}\sum_{i=1}^{N}y_{ij}log{\overset{ˆ}{y}}_{ij}+(1-y_{ij})log(1-{\overset{ˆ}{y}}_{ij})$$

where $y_{ij}\in \{0,1\}$ indicates the actual interaction label of the drug pair $\left(d_{i},d_{j}\right)$ in the binary classification task, and ${\overset{ˆ}{y}}_{ij}$ represents the predicted DDI probability.

## 3 Experiments and result analysis

Our experiments are implemented using the Pytorch framework, while molecular graph conversions are handled by RDKit [38]. The parameters are configured as follows: the learning rate is established at $1\times 10^{-4}$, batch size at 16, and the training extends over 50 epochs. The sequence encoder utilizes a Transformer with 8 attention heads, outputting a feature vector length of 75. The graph encoder, designed with a message vector size of 25, processes information through 2 layers of message passing, culminating in a 2D graph feature vector of length 75. The third encoder comprises 3 attention block layers, 8 attention heads, and a hidden dimension of 256 for drug encoding. Optimization leverages the Adam optimizer, and the binary cross entropy loss function is utilized to enhance model performance.

### 3.1 Datasets and evaluation metrics

We employ three DDIs datasets: Pang et al. dataset [40], BioSNAP [41], and AdverseDDI [42]. Pang et al. dataset is derived from Drugbank [43], which comprises 1548 drugs and 34282 DDI relationships, the BioSNAP dataset includes 1,306 drugs with 41,010 labeled DDIs, and the AdverseDDI dataset includes 388 drugs with 12,288 labeled DDIs. We maintain a balanced ratio of positive to negative samples. In the three datasets, each sample pairs two drugs with a label (1 for presence, 0 for absence of an interaction). The dataset is partitioned randomly into training, testing, and validation sets with proportions of 8:1:1, respectively, to train and assess our approach.

Our evaluation employs three metrics: accuracy (ACC), area under the ROC curve (AUROC), F1-score. To mitigate the effects of random variability and ensure the robustness of our results, we conduct each experiment five times. The final results are presented as the average values from these repeated experiments.

### 3.2 Baselines

In comparative experiments, we evaluate our method against several baseline methods. These include Random forest (RFC) [44], Deepconv [9] and Long Short-Term Memory (LSTM) [45], which are traditional networks designed for processing sequence information. Additionally, we compare against graph-based classification model like Graph Attention Network (GAT) [46]. The above methods are DDI prediction methods based on single perspective, either sequence or molecular graph. While AMDE [40], DNS-DDI [47] and HTCL-DDI [31] are methods of feature learning for DDI prediction by using two or triple perspectives.

(1) RFC [44], a machine learning classifier, processes high-dimensional vectors formed by concatenating Morgan fingerprint pairs of drugs, efficiently handling large datasets without dimensionality reduction.

(2) Deepconv [9] uses Morgan fingerprint as input. It extracts drug features through DNN. The features of drug pairs are concatenated as input to a dense layer to predict DDI outcome.

(3) LSTM [45] combats gradient issues in long sequence training by encoding Morgan fingerprints and passing the resultant features through a fully connected layer for DDI prediction.

(4) GAT [46] extracts features using a Graph Convolutional Network (GCN) with eight attention heads, then predicts DDIs via a feedforward network.

(5) DeepDDI [7] forms a drug similarity matrix from fingerprints, reduces dimensions with PCA, and predicts interactions via a deep neural network.

(6) DeepDDS [48] used SMILES sequences to extract drug representations, calculated the similarities between drugs, and subsequently employed deep neural networks for prediction.

(7) AMDE [40] fuses features from SMILES sequences and drug graph structures for DDI prediction.

(8) DSN-DDI [47] adopts a dual-view approach to learn drug substructure representations, using features to predict DDIs with three linear layers.

(9) HTCL-DDI [31] employs multidimensional feature extraction: molecular, structural, and semantic to model complex DDI information.

### 3.3 Overall performance

Table 1 presents the averaged results for MFE-DDI and other baseline methods on the three datasets. The data shows that our approach outperforms others in metrics of F1-score, Accuracy (Acc), and Area Under the Receiver Operating Characteristic Curve (AUROC) on the three datasets, indicating superior performance.

**Table 1:** Comparison Results (Mean Scores in %).

Model	Pang et al.			BioSNAP			AdverseDDI		
	F1	ACC	AUROC	F1	ACC	AUROC	F1	ACC	AUROC
RFC	79.94	81.19	89.60	74.80	74.34	82.57	77.17	75.42	83.60
DeepDDI	81.43	90.44	92.18	88.60	89.23	94.74	87.26	87.45	94.34
DeepDDS	89.05	88.65	94.86	87.55	87.93	95.21	82.45	85.34	93.07
Deepconv	97.19	97.30	98.61	97.70	97.70	99.14	88.65	88.74	95.21
LSTM	96.35	96.14	98.49	97.75	97.72	98.78	88.41	88.23	94.30
GAT	95.87	95.39	97.30	91.19	91.52	96.82	86.14	85.92	93.62
AMDE	97.33	96.68	98.90	97.37	97.38	99.19	86.57	86.33	93.54
HTCL-DDI	96.33	96.34	95.99	-	-	-	85.07	85.27	92.14
DSN-DDI	96.90	96.80	98.28	93.72	93.55	97.89	83.76	84.13	92.62
**MFE-DDI**	**97.91**	**97.87**	**99.18**	**98.20**	**98.23**	**99.22**	**89.52**	**89.38**	**95.62**

Due to equipment memory limitation, we were unable to run HTCL-DDI on BioSNAP dataset.

As a multidimensional feature learning framework, MFE-DDI surpasses all single-perspective baseline methods, highlighting the benefits of incorporating multi-perspective feature learning into DDI prediction. MFE-DDI examines the same drug from various perspectives, comprehensively utilizing feature information from multiple views, including SMILES sequence features, molecular graph features, and atomic space semantic features. By fusing these diverse features, it achieves a more reliable and comprehensive drug representation. Furthermore, MFE-DDI surpasses other multi-perspective feature learning frameworks on the three datasets, showcasing its superior potential in DDI prediction. This highlights MFE-DDI's ability to capture key features related to DDI through multi-perspective feature learning.

### 3.4 Robustness analysis

In order to analyze the robustness of MFE-DDI, in this section, we performed anti-perturbation assessment of our method on Pang et al. dataset by gradually reducing percentage of 25%, 50% and 75% from the dataset. At the same time, we select four baseline methods including DeepDDS, HTCL-DDI, DeepDDI and DSN-DDI for comparison and evaluation.

**Fig. 2:**
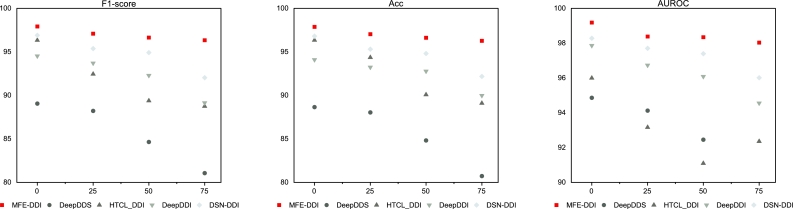
Robustness analysis by gradually reducing percentage of 25%, 50% and 75% from the dataset.

The results are shown in Fig. 2. It can be seen that our method still achieves the best results in the case of data reduction. Compared with the full dataset, the scores of our method only decrease by less than 2%. For other methods, these metrics change drastically when the data scale is reduced. Their performance depends heavily on large samples and rich instances, and performs poorly on small-sample datasets. MFE-DDI achieves this robust performance by directly encoding molecular-level characteristics rather than DDI network-derived patterns. Specifically, SMILES sequences encoding captures the contextual relationship between functional groups, and the molecular graph structure intuitively reflects the topological characteristics of drugs, along with a more detailed semantic encoding of the spatial conformation of atoms and bonds. They each focus on different aspects of the exploration of drug molecular properties, and overall, the features complement each other effectively after integration. This contributes to a more robust and comprehensive representation of drugs compared to other methods, which can explain the robustness performance of MFE-DDI as the decreasing of dataset scale.

### 3.5 Ablation studies

The superiority of MFE-DDI is largely due to the introduction of SMILES sequence information, molecular graph structure information and atomic spatial semantic information into the DDI prediction. To explore their impact on performance, we conduct ablation studies on the feature encoders of the three channels.

**MFE-DDI** has the feature encoder that combines three types of information, including SIMLES information, molecular graph information and atom spatial semantic information. The overall feature is fused based on attention mechanism.

**no_seq** means the feature encoder does not contain SMILES sequential information.

**no_graph** means the feature encoder does not contain molecular graph information.

**no_semantic** means the feature encoder does not contain atom spatial semantic information.

**seq_only** means the feature encoder only contains SMILES sequential information.

**graph_only** means the feature encoder only contains molecular graph information.

**semantic_only** means the feature encoder only contains atom spatial semantic information.

In the above, **no_seq**, **no_graph** and **no_semantic** are dual-view models, while **seq_only**, **graph_only** and **semantic_only** are single-view models.

As shown in Table 2, removing any module degrades the model's overall performance. Specifically, the performance of graph_only model, which achieves the highest scores among single-view models, highlights the pivotal role of molecular graph structural characteristics in DDI prediction. This is attributable to the clear representation of the connectivity and bond types between atoms in the molecular structure, directly reflecting the topological characteristics of drugs.

**Table 2:** Ablation Analysis Results.

Methods	sequence	graph	semantic	F1-score	ACC	AUROC
MFE-DDI	✓	✓	✓	97.91	97.87	99.18
no_seq	-	✓	✓	97.19	97.12	98.91
no_graph	✓	-	✓	94.32	91.21	98.73
no_semantic	✓	✓	-	97.34	96.82	98.70
seq_only	✓	-	-	92.76	88.98	98.89
graph_only	-	✓	-	96.56	96.48	97.98
semantic_only	-	-	✓	96.39	96.31	97.80

Incorporating SMILES information (no_semantic model) further improves performance. Actually, by utilizing SMILES sequence information, it is possible to capture the contextual relationships between functional groups within the sequences. The feature of sequences encoded from SMILES can provide discriminant capabilities that are complementary to molecular graph.

Finally, our full model, which further integrates atomic-level spatial semantic features, achieves a more enhanced performance. This underscores the importance of finer-grained atomic spatial features in capturing nuanced drug-drug interactions. A more detailed encoding of the spatial positions of atoms and bonds results in semantic features that play a crucial role in determining the properties of drugs.

Overall, our model approach achieved the best performance, effectively illustrating that the combination of three feature encoding channels enables MFE-DDI to enhance the perception of drug essential information.

### 3.6 Sensitivity analysis

**Fig. 3:**
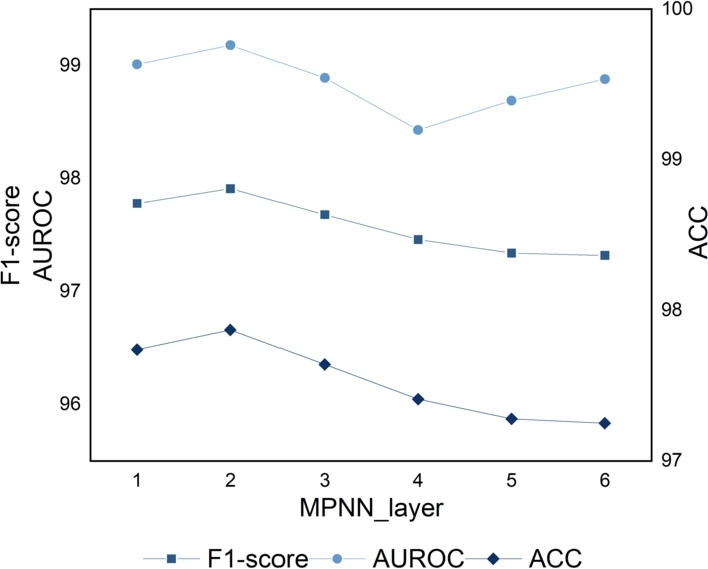
Sensitivity analysis on MP-num.

In this section, sensitivity analysis is conducted on the message passing parameters (MP-num) used in the molecular graph feature extraction module. In order to find out the hyperparameters that can achieve the best performance of our model, we assign different values to MP-num and then evaluate the performance of MFE-DDI on the Pang et al. dataset. We picture the performance changes of MFE-DDI when MP-num = {1, 2, 3, 4, 5, 6} in Fig. 3.

Results show that increasing MP-num beyond a certain point leads to decreased performance. Moreover, the larger the MP-num is, the computational consumption of our method will simultaneously increase. The optimal setting, MP-num = 2, yields the highest scores.

### 3.7 Case study

In this section, we selected three drugs to evaluate the generalization ability and potential predictive power of our proposed MFE-DDI method.

Following the methodology of PTB [20], Resmetirom [49] and Mavorixafor [50], approved by the Food and Drug Administration (FDA) in 2024, are selected for evaluation firstly. Resmetirom is the first FDA-approved oral THR-*β* agonist for noncirrhotic NASH with moderate-to-advanced liver fibrosis, targeting hepatic lipid metabolism and fibrotic pathways. Mavorixafor is a first-in-class CXCR4 antagonist approved for WHIM syndrome, addressing symptoms of warts, hypogammaglobulinemia, infections and myelokathexis via CXCL12/CXCR4 axis modulation.

Since the two drugs did not appear in the previous dataset, obtaining their true interaction labels with other drugs was challenging. Therefore, we validated the model using randomly generated drug pairs containing these two drugs. The output of the model is the interaction probability (0-1) of drug pair, the closer the prediction result is to 1, the more likely it is that the two drugs will interact. Table 3 lists the potential DDIs predicted by our model, along with their corresponding prediction scores and interaction descriptions sourced from DrugBank [43].

**Table 3:** Predicted Drug-Drug Interactions with Resmetirom and Mavorixafor.

New Approved Drugs	DrugBank ID1	Interacting Drugs	DrugBank ID2	Score	Interaction Description
Resmetirom	DB12914	Atorvastatin	DB01076	0.975	The serum concentration of Atorvastatin can be increased.
		Rosuvastatin	DB01098	0.601	The serum concentration of Rosuvastatin can be increased.
		Pitavastatin	DB08860	0.954	The serum concentration of Pitavastatin can be increased.
		Nefazodone	DB01149	0.948	The serum concentration of Resmetirom can be increased.
		Lovastatin	DB00227	0.981	The serum concentration of Lovastatin can be increased.

Mavorixafor	DB05501	Mirtazapine	DB00370	0.868	The risk or severity of QTc prolongation can be increased.
		Fluvastatin	DB01095	0.876	The metabolism of Fluvastatin can be decreased.
		Paliperidone	DB01267	0.838	The excretion of Mavorixafor can be decreased.
		Asenapine	DB06216	0.897	The risk or severity of QTc prolongation can be increased.
		Desvenlafaxine	DB06700	0.908	The metabolism of Mavorixafor can be decreased.

Besides, we take Pseudoephedrine (DB00852) [43] as an example and use our MFE-DDI model to predict drugs that may interact with it. The result and analysis can be seen in Supplementary 1. The successful predictions of these potential DDIs demonstrate the predictive power of our method.

## 4 Conclusion

In this study, we developed the MFE-DDI, a drug-drug interactions (DDIs) prediction model, based on a multidimensional feature learning framework. This model simultaneously encodes the multidimensional characteristics of drugs from three perspectives: the SMILES sequence of drugs, molecular graph, and atomic space semantic information. We evaluate the performance of MFE-DDI on three datasets, which indicate that our method surpasses other baselines. Moreover, more experiments are conducted to demonstrate the robustness of the model and the necessity of each component of the model. Case studies on multiple drugs demonstrate the potential of our model in predicting unknown drug-drug interactions. All experimental results show that multidimensional features have a powerful representation capability in DDI prediction tasks, and MFE-DDI can provide an effective computational tool for DDI prediction.

## Funding

This article is sponsored by National High Level Hospital Clinical Research Funding, 2023-NHLHCRF-YXHZ-ZRZD-02; the 10.13039/501100005089Natural Science Foundation of Beijing Municipal No. 4252021, 10.13039/501100001809National Natural Science Foundation of China, No. 62272009, 62376017; 10.13039/501100012226Fundamental Research Funds for the Central Universities buctrc202403, buctrc202221. The first and the second affiliations are of equal status.

## CRediT authorship contribution statement

**Lingfeng Wang:** Supervision, Project administration. **Yinghong Li:** Writing – original draft, Methodology, Investigation. **Yaozheng Zhou:** Validation, Formal analysis. **Liping Guo:** Funding acquisition. **Congzhou Chen:** Methodology, Funding acquisition, Conceptualization.

## Declaration of Competing Interest

The authors declare that they have no known competing financial interests or personal relationships that could have appeared to influence the work reported in this paper.

## Appendix A Supplementary material

MMC Case Study on Drug Pseudoephedrine (DB00852). MMC
