# Supplementary material for: MFE-DDI: A multi-view feature encoding framework for drug-drug interaction prediction
Source: Comput Struct Biotechnol J. 2025 May 26;27:2473–80. doi: 10.1016/j.csbj.2025.05.029 (PMC12181007; doi:10.1016/j.csbj.2025.05.029)
Supplement: MMC — Case Study on Drug Pseudoephedrine (DB00852). [file mmc1.docx]

Pseudoephedrine (DB00852) is an α and β adrenergic agonist that stimulates sympathetic nerve endings to release norepinephrine to constrict blood vessels. Since the drug mainly constricts blood vessels in the upper respiratory tract, it is clinically used to treat nasal and sinus congestion and allergic rhinitis. We take Pseudoephedrine (DB00852) as an example and use our MFE-DDI model to predict drugs that may interact with it. In Table S1, we list 15 high-scoring drugs with prediction results greater than 0.95, which also include new drug-drug interactions that do not appear in the training process. Meanwhile, these drugs have all been demonstrated to interact with Pseudoephedrine (DB00852). Among these drugs, Tranylcypromine and Phenelzine are both monoamine oxidase inhibitors. They inhibit the action of related hormones by restraining the decomposition of neurotransmitters such as adrenaline and increase the concentration and activity of neurotransmitters in the nervous system, thus causing antidepressant and anti-anxiety pharmacological effect. Monoamine oxidase inhibitors counteract Pseudoephedrine (DB00852) in their mechanism.

**Table S1. Case Study on Drug Pseudoephedrine (DB00852).**

|  | Drug name | Drugbank ID | Verification source | True_Label | Prediction_Score |
| --- | --- | --- | --- | --- | --- |
| 1 | Tranylcypromine | DB00752 | Drugbank | 1 | 0.9987 |
| 2 | Phenelzine | DB00780 | Drugbank | 1 | 0.9975 |
| 3 | Chlorpromazine | DB00477 | Drugbank | 1 | 0.9908 |
| 4 | Thioridazine | DB00679 | Drugbank | 1 | 0.9980 |
| 5 | Trifluoperazine | DB00831 | Drugbank | 1 | 0.9951 |
| 6 | Olanzapine | DB00334 | Drugbank | 1 | 0.9914 |
| 7 | Asenapine | DB06216 | Drugbank | 1 | 0.8910 |
| 8 | Lurasidone | DB08815 | Drugbank | 1 | 0.9719 |
| 9 | Iloperidone | DB04946 | Drugbank | 1 | 0.9969 |
| 10 | Paraldehyde | DB09117 | Drugbank | 1 | 0.9973 |
| 11 | Thiothixene | DB01623 | Drugbank | 1 | 0.9927 |
| 12 | Loxapine | DB00408 | Drugbank | 1 | 0.9571 |
| 13 | Duloxetine | DB00476 | Drugbank | 1 | 0.9884 |
| 14 | Levomilnacipran | DB08918 | Drugbank | 1 | 0.9964 |
| 15 | Venlafaxine | DB00285 | Drugbank | 1 | 0.9985 |
